# A solitary bronchial papilloma with unusual endoscopic presentation: case study and literature review

**DOI:** 10.1186/1471-2466-9-40

**Published:** 2009-08-18

**Authors:** Fabrice Paganin, Martine Prevot, Jean Baptiste Noel, Marie Frejeville, Claude Arvin-Berod, Arnaud Bourdin

**Affiliations:** 1 Service de Pneumologie. GHSR. St Pierre. France; 2 Service d'Anatomo-Pathologie. GHSR. St Pierre. France; 3 Service de Radiologie. GHSR. St Pierre. France; 4 Service de Pneumologie. Hôpital Arnaud de Villeneuve. Montpellier. France

## Abstract

**Background:**

Solitary endobronchial papillomas (SEP) are rare tumors and most of them are described by case report. A misdiagnosis is common with viral related papillomas. A histopathological classification has recently permitted a major advancement in the understanding of the disease.

**Case Presentation:**

We report a case of a mixed bronchial papilloma with an unusual endoscopic presentation. The literature was extensively reviewed to ascertain the unusual characteristics of the current case. A 39-year of age male was referred to our institution for the investigation of a slight hemoptysis. Routine examination was normal. A fibroscopy revealed an unusual feature of the right main bronchus. The lesion was a plane, non-bleeding, non-glistering sub-mucosal proliferation. No enhanced coloration was noticed. Biopsies revealed a mixed solitary bronchial papilloma. In situ HPV hybridization was negative. Endoscopic treatment (electrocautery) was effective with no relapse.

**Conclusion:**

This lesion contrasts with the data of the literature where papilloma were described as wart-like lesions or cauliflower tumors, with symptoms generally related to bronchial obstruction. We advise chest physicians to be cautious with unusually small swollen lesions of the bronchi that may reveal a solitary bronchial papilloma. Endoscopic imaging can significantly contribute to the difficult diagnosis of SEP by pulmonary physicians and endoscopists.

## Background

Solitary endobronchial papillomas (SEP) are rare tumors. To date, less than 50 cases were described. Due to low frequency, the majority of data available in the literature are case reports. A major advancement was achieved in 1999 with a large histopathological review that led to a classification of the different sub-types related to solitary papilloma [1]. An another important issue in the management of SEP is the selection of therapy. Surgical resection was the gold standard, but SEP being considered as benign tumors, endobronchial treatment was successfully used (YAG Laser, electrocautery). We are reporting a case of SEP we treated with endobronchial electrocautery, which had unusual clinical and endoscopic properties.

Due to the rarity of SEP, we are reporting here a single case. The history of the patient was carefully analyzed. Extensive review of the published literature was made to compare this uncommon clinical and endoscopic presentation to generally described features.

## Case presentation

**Figure 1:**
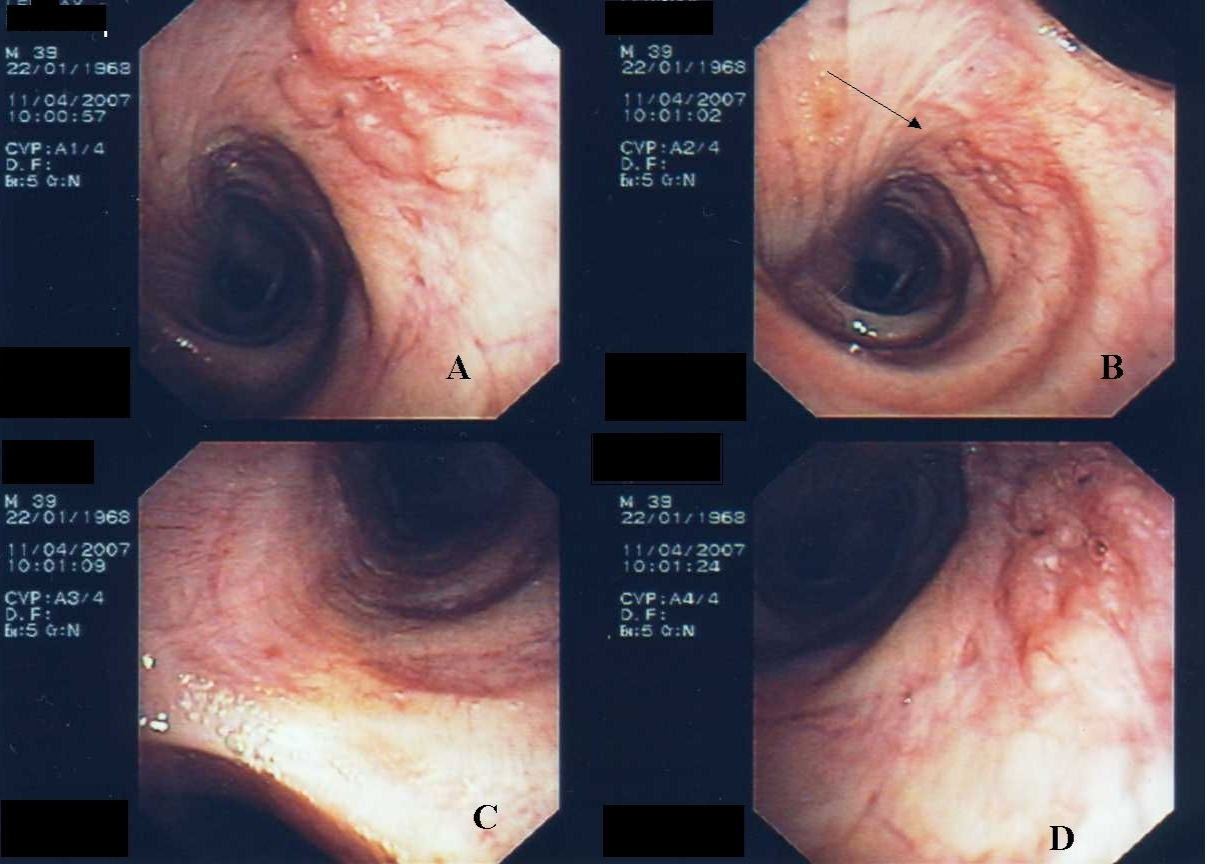
**Bronchoscopic features at admission**. Right main bronchus with a plane non-glistering sub-mucosal lesion (panel A, D). The lesion (arrow) may be missed in non-optimal endoscopic conditions (panel B). The carina and the left main bronchus were normal (panel C).

**Figure 2:**
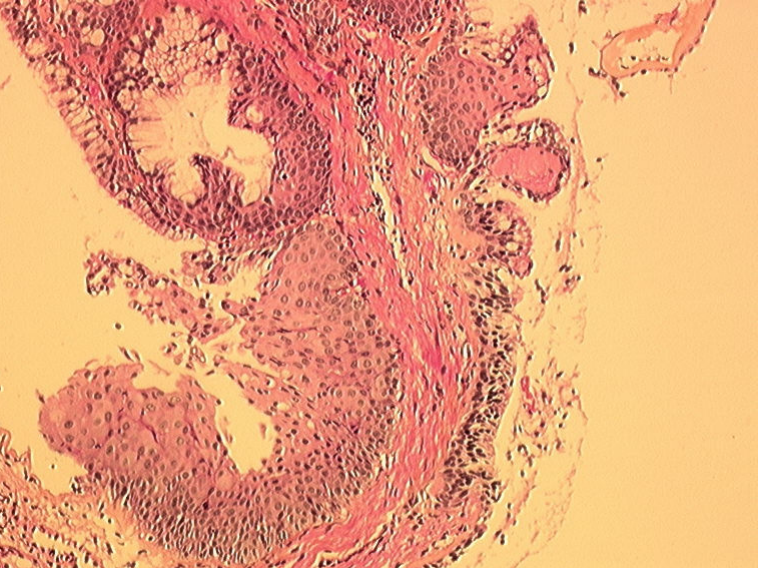
**Mixed solitary bronchial papilloma**. At this magnification, fibrovascular cores are lined by squamous and glandular epithelium. Hematoxylin-eosin stain; magnification × 100.

A 39-year old man was referred to our institution for investigation of recent cough. He experienced a unique slightly bloody sputum. He was a mild current smoker (less than 15 packs/year). There were no baseline abnormal conditions, and physical examination was unremarkable. The chest-X-Ray was normal. The bronchoscopy revealed a swollen lesion at the internal proximal part of the main right bronchus (figure 1). Endoscopic findings were uncommon. The entire lesion measured less than 1 cm^2^. It was a plane, non-bleeding, non-glistering sub-mucosal lesion. No enhanced coloration appeared. The tumor was soft and biopsies were easy despite the tangential localization in the bronchus. Histopathological examination revealed a mixed papilloma. It was an endobronchial polypoid lesion, 2.5 cm long and 0.2 cm wide, made of thin fibrovascular cores with moderate lymphoplasmocytic infiltrates, and lined by squamous and glandular epithelium. Pseudo-stratified ciliated and non ciliated, cuboidal to columnar cells, with rare mucinfilled cells were mixed with acanthotic and focally keratinizing squamous epithelium. Squamous atypia, ranging from mild to severe dysplasia could be seen, but viral cytopathic changes were not reported. No glandular atypia nor necrosis were seen (figure 2). In situ HPV hybridization was negative.

The CT scan did not find any abnormalities: absence of significant lymph nodes, no bronchial thickening, no peripheral mass or nodules. Pulmonary function tests were normal. Serum tumors markers (CEA, SCC antigen, CYPHRA 21-1) were also normal.

Endoscopic treatment was proposed with a rigid bronchoscope (EFER, La Ciotat, France) under general anesthesia. We used an electrocautery knife (ERBE, Tübingen, Germany). The procedure was rapid and easy. A fiberoptic bronchoscopy was performed 7 days after the procedure showing a total destruction of the tumor with a small scar. Further endoscopic controls over 12 and 24 months did not reveal any relapse and the patient was considered to be cured.

## Conclusion

This case produced two interesting findings. First, solitary bronchial papillomas are rare tumors and the description of a new case enhances the understanding of this disease. Second, the endoscopic presentation of this mixed solitary papilloma is uncommon and contrasts with previous descriptions in the literature.

Papillomas were recently classified and subdivided into 3 categories: 1 – squamous cell, 2 – glandular and 3 – mixed [1]. Solitary squamous papillomas are seen predominantly in middle-age men, with generally a past or current history of tobacco smoking. Glandular and mixed papillomas have a different presentation. They occur in older men and women and are less related to tobacco smoking [2]. A major issue for squamous papilloma is its potential malignancy, as previously reported [3]. Fliedler et al. reported the difficulty to to grade dysplasia in squamous papillomas due to their epithelial architectural distortion, inflammation, and occasional koilocytic changes [1]. Malignancy was described in Japanese case reports [3-5] but the exact relationship between solitary papillomas and bronchogenic carcinomas remains unclear. Preexisting lesions may evolve towards polypoid squamous cell carcinomas but this is considered as extremely rare. The pathogenetic role of HPV also remains unclear. Cytopathogen effects were found in SEP in patients with condylomatous papillomas [6]. Katial et al. found HPV DNA in a solitary squamous papilloma [7]. They hypothesized that the HPV virus was acquired through aspiration of infected secretions in a sexually active young men. For SEP, it is possible that HPV plays a role in the development of SEP but not alone [8]. HPV DNA in situ hybridization failed to be positive in the majority of SEPs evaluated by Flieder et al. [1]. Human papillomavirus appears to play a pathogenic role in some squamous cell papillomas, but not in mixed papillomas [1,9].

It is important to differentiate closed related entities. Condylomatous papillomas are related to viral infection and, may occasionally be found in larynx or airways [10]. However, the history of these patients is extremely different from that of patients with SE*P*, as it includes risk factors related to viral infection [11,12] For adults a sexually-transmitted disease was possible. Some papillomas were also associated with inhaled foreign bodies [1,13]. Barzo et al. found papillomas associated with bone chicken impacted in the bronchus. Another cause was broncholithiasis. These papilloma-like structures were inflammatory patterns due to foreign bodies. SEP induced by foreign bodies and broncholithiasis, like viral ones have to be excluded of the SEP series as emphasized by Fliedler (exclusion of 100 examples of so-called solitary papillomas after critical review and, analysis of 27 definite cases) [1].

A major issue in SEP is the clinical presentation. The analysis of the literature shows protean symptoms and chest-X-Ray presentation. The 8 Japanese cases found 5/8 (63%) patients with cough and wheezing [3]. The 3 remaining patients were non-symptomatic. Cough and asthma-like symptoms were common. Others symptoms were more related to bronchial obstruction with recurrent pneumonia and lobar collapse. Hemoptysis was reported in 4/13 patients [1]. Our patient had minor bleeding sputum, but this symptom was considered as hemoptysis. However, we did not find any blood trace in the airways and the tumor was not bleeding. Macroscopic examination during fibroscopy did not reveal any abnormal vasculature. Radiographic features were also variable. Among the 8 patients in the Japanese series, one only had a normal chest-X-Ray, the 7 others had abnormalities ranging from infiltrative shadow to hilar mass and collapse [3]. The analysis of several case reports revealed abnormal shadows on chest-X-Rays for 6 patients [14-19] and no abnormalities for 1 [4]. In his series of 13 patients, Flieder found 3/5 radiographic abnormalities for solitary squamous papillomas, 2/3 for glandular papillomas and 4/5 for mixed papillomas [1]. Focal bronchiectasis was a relatively common radiographic feature and was reported in a case report [7] and in 2 cases of the Flieder series [1].

We have little information on the endoscopic features for SEP. As the majority of SEP presented with radiographic abnormalities highly suggestive of a tumoral disease, bronchoscopy was the first diagnostic tool used. In 1965, Drennan and Douglas described a solitary papilloma of the bronchus (2 × 1.5 cm), but did not find any bronchoscopic abnormalities [18]. This was surprising as the chest-X-Ray localized the tumor at the origin of the left lower lobe. At least, the patient experienced a coughing up of a pedunculated tumor. More recent reports found various endobronchial abnormalities. Inoue et al. described a polypoid tumor obstructing the middle lobe [3]. Kattial et al. found a bronchial stenosis of a left upper lobe segment, but SEP was located below the stenosis and a definite diagnosis was obtained after surgery [7].

All the lesions described by Fliedler et al. were similar: polypoid, friable tan to red, glistering and, ranged in size from 0.2 to 2.5 cm. No significant differences were seen between histologic sub-types [1]. An extensive description of 8 bronchial papillomas was published more than 20 years ago [13]. Endoscopic localization was presented and confirmed the fact that SEP are located in the proximal part of the airways. SEP were described as peduncular tumors but Barzo et al. described 5/8 tumors with a large seated base with a sharp border. They compared them to a raspberry or blackberry with a papillary structure, even to a cauliflower. All tumors were seen as tumor-like wart [3,4,13]. Others endoscopic descriptions were available. Both retrieved white, soft, cauliflower-like tumors obstructing the bronchial lumen [4,19].

It is difficult to compare our findings to the literature, as our findings seem to be unique. Our patient did not present with a common tumor. It was not a peduncule-like tumor but an infiltrative mode. The lesions arose the bronchus wall but cannot be considered as wart-like with normal bronchus mucosa coloration. Obviously, as shown on figure 1 (arrow, panel B), this lesion can be easily missed by an endoscopist with limited experience. SEP occurred generally in the middle-aged to aged population. Aging patients have non-pathological endoscopic modification with atrophia, increase of gland ducts, cartilaginous strictures. Reorganization of the bronchial tree is more intense in chronic obstructive pulmonary disease (COPD) patients. Fortunately, our patient was young, had no COPD underlying conditions and the lesion was located just above the carina. The same lesion located in a segmental bronchus in a COPD patient will be certainly more difficult to identify.

Endoscopic treatment was proposed here. Considering the absence of abnormalities on CT-scan examination and the small size of the tumor, this decision was acceptable. YAG-Laser therapy is commonly used [3]. We preferred to use an electrocautery knife because of its low perforation risk compared to high energy Laser. Recurrence was observed in the literature after endoscopic resection but re-excision through endoscope was possible and efficient after a 16-years disease-free period [1]. To date, 1 year after the electrocautery procedure, the patient remains asymptomatic with a normal endoscopic control.

In conclusion SEP are rare tumors. It is currently important to discriminate real solitary pulmonary papillomas from others related papillomas and achieve to obtain a definite histological sub-type. Clinical, radiographic and endoscopic presentations are generally more related with usual tumor management. We advise chest physicians to be cautious with unusually small swollen lesions as described here, as they could relate to SEP. Biopsies are easy, and contrast with the difficulty of the pathologic examination. In case of a limited lesion, endoscopic therapy is mandatory.

## Abbreviations

**SEP**: Solitary endobronchial papillomas; **CEA**: carcino embryonary antigen; **SCC**: squamous cell carcinoma; **HPV**: human papilloma virus; **COPD**: chronic obstructive pulmonary disease; **CT**-scan: computed tomography scanner.

## Competing interests

The authors declare that they have no competing interests.

## Authors' contributions

FP, CAB and AB designed and wrote the manuscript. JBN analyzed the radiologic data and reviewed the manuscript. MP and MF wrote the pathologic section and reviewed the manuscript.

## Consent

Written informed consent was obtained from the patient for publication of this case report and any accompanying images. A copy of the written consent is available for review by the editor-in-chief of this journal

## Pre-publication history

The pre-publication history for this paper can be accessed here:


